# Effects of Sport Teaching on Students' Enjoyment and Fun: A Systematic Review and Meta-Analysis

**DOI:** 10.3389/fpsyg.2021.708155

**Published:** 2021-08-04

**Authors:** Manuel Rodríguez Macías, Manuel Tomás Abad Robles, Francisco Javier Giménez Fuentes-Guerra

**Affiliations:** Faculty of Education, Psychology and Sport Sciences, University of Huelva, Huelva, Spain

**Keywords:** physical education, sport pedagogy, sport teaching models, school sport, sport initiation

## Abstract

The methodology used in sport teaching influences not only the students' technical and tactical learning, but also psychological and social dimensions such as enjoyment. We aimed to analyze the effects of interventions using conventional and non-conventional sport teaching methodology on students' enjoyment/fun, through a systematic review and meta-analysis. The search was carried out following the PRISMA Statement in the databases of Web of Science, PubMed, Scopus, SportDiscus, Eric and PsycInfo. A total of 1,481 documents were obtained, with the addition of 5 more which were identified in the reference lists of the articles found in the databases. Eleven articles were considered to meet the inclusion criteria. The meta-analysis yielded a moderate effect size of 0.72, and a 95% CI from 0.48 to 0.96 in favor of non-conventional teaching methods, highlighting the model of sports education. Nevertheless, the heterogeneity of the interventions was large and the quality of the evidence, according to GRADE, was very low. In conclusion, the use of non-conventional models in sport teaching with the aim of increasing the enjoyment/fun of boys and girls is advised. These suggestions could be useful for teachers and sport coaches to increase the enjoyment/fun of their trainees during sport practice.

## Introduction

Over the past two decades, the Attainment Goals Theory (Nicholls, 1989) together with the Self-Determination Theory (Deci and Ryan, 2000) and Vallerand's (2001) Hierarchical Motivation Model have examined the motivational process of physical education students, highlighting that the interpersonal style of the teacher/coach may influence this process. It is known that during adolescence there is a significant decrease in the motivation and participation of boys and girls (Sallis and McKenzie, 1991). This issue is important when boys and girls learn to play a sport (sport initiation), in physical education classes. At this stage of training it is essential that boys and girls have fun practicing sports, as this will facilitate adherence (Ortega et al., 2014). Therefore, the teacher faces the difficult challenge of getting their students to enjoy and have fun in physical education. This question is essential, since enjoyment/fun are fundamental aspects of learning (Baena-Extremera et al., 2012b). In this sense, sport can be a means of great value, as it is often seen as a facilitator of enjoyment and fun in boys and girls. On the other hand, although enjoyment/fun and motivation are closely related, they are not concepts meaning the same thing (Wiersma, 2001). In this way, Scanlan and Lewthwaite (1986) and Fairclough (2003) consider that enjoyment/fun differs from motivation and should therefore be seen as a broader and more inclusive construct. In addition, enjoyment/fun has also been associated with intrinsic motivation (e.g., feeling of affiliation with peers, positive social interactions), with extrinsic aspects (e.g., competition, social recognition of sports achievement) and with a flow state (Wiersma, 2001). Moreno et al. (2009) define enjoyment/fun as the meaning people give to the activities performed, while motivation, according to Valle et al. (1997), is what makes an individual decide to do something in order to achieve a goal he or she has set for him or herself. Enjoyment is “a positive affective response to the sport experience that reflects generalized feelings such as pleasure, liking, and fun” (Scanlan et al., 1993, p. 6). Accordingly, Harwood et al. (2008) state that the individual goals students set for themselves can affect their motivation, effort, enjoyment, and level of physical activity. Sometimes there is a tendency to differentiate between play and learning, which is why teachers may sometimes consider motivation and enjoyment/fun to be unrelated (Gros, 2009). However, as Ntoumanis (2005) believes, when individuals have fun, they tend to be intrinsically motivated and tend to attribute more importance to the subject matter and, as a consequence, they learn more. In this sense, enjoyment gives rise to a positive reaction to participation, and may enable researchers and practitioners to ensure a positive experience for young athletes (Wiersma, 2001).

Similarly, Hassinger-Das et al. (2017) consider enjoyment/fun to be a key element in engaging children, maintaining their interest, and enhancing their learning, while Baena-Extremera et al. (2012b) state that motivation, satisfaction, and fun are crucial in order to learn. Along these lines, Riera (2010) claims that the characteristics of the tasks, the functionality of the student, the dispositional factors and the information conveyed are factors influencing the student's enjoyment/fun when learning a particular sport. For this reason, Alarcón et al. (2010) consider that the tasks should be sequences in an ascending progression in difficulty, as this facilitates the learning of a certain skill, which helps to avoid frustration and boredom of students. Accordingly, Baena-Extremera et al. (2016) consider that it is essential for students to enjoy and feel satisfied with Physical Education class, since the intention to practice sport is usually related to enjoyment/fun (Moriana et al., 2006). In this vein, studies carried out by Côté (1999), and Côté and Hay (2002) reveal that enjoyment/fun is a vital determinant of long-term sport participation. In addition, research such as Cairney et al. (2012), Leptokaridou et al. (2015), and Jaakkola et al. (2017) show that higher levels of enjoyment/fun in physical education classes lead to more motivated and engaged participation, and a significant increase in regular physical activity outside of school.

Sport is often seen as an element of fun and enjoyment. However, it is common to see participants put more emphasis on winning than on developing values such as fun, which may be due to the teaching model used (Carron and Brawley, 2008). In this way, scientific literature indicates that the methodology used in the sport teaching influences not only technical and tactical learning, but also psychological and social dimensions such as enjoyment/fun (Emmanouel et al., 1992). Ortega et al. (2008) believe that teachers and coaches should try to make activities fun and avoid those which are not, while Cecchini et al. (2014) assert that enjoyment/fun is increased when teachers promote active classes where students feel that they are the protagonists. However, conventional models are characterized by the single mandate of the teacher/coach, which obstructs the autonomy of athletes and, as a consequence, generates anxiety, boredom and even undisciplined behavior (Reeve and Tseng, 2011). Conventional or traditional approaches to sports education focus on a perfect model of technical performance that boys and girls practice repeatedly and in isolation, through non-contextualized tasks different from the real game (Blomquist et al., 2001) and through direct instruction. In these approaches, boys and girls do not feel protagonists of their learning, and enjoyment/fun may decrease (García-López et al., 2012). In this sense, non-conventional approaches are based on constructivist theory where students are seen as creative, social, and active learners who build their own knowledge and identify what they need to improve during the learning process (Dyson et al., 2004). These approaches emerge as a reaction to conventional models and focus on the simultaneous development of technique, tactical knowledge and decision-making using real game-like situations, and through problem-solving, e.g., Teaching Games for Understanding (TGfU), Sport Education Model (SE), and Cooperative Teaching. In addition, these pedagogical models promote opportunities for both boys and girls to solve problems, make decisions and demonstrate leadership (Metzler, 2011). Thus, Mouratidis et al. (2011) state that in Physical Education classes there are differences in terms of student satisfaction and enjoyment/fun depending on the way in which the teacher approaches the content. Following this line, the results obtained by Burgueño et al. (2016), reveal that the enjoyment/fun of the group receiving Physical Education classes through a non-conventional model increased, while it decreased in those who received traditional teaching. It seems advisable to use non-conventional models in sport teaching with the aim of increasing the enjoyment of students (Batez et al., 2021). Other studies have reported that the reduction of sport practice during free time by students may be related to the use of traditional teaching methodology, because teaching situations are presented in a decontextualised way (Crance et al., 2013), which may generate boredom in boys and girls, and may be far from their motivations (García-López et al., 2012). Likewise, Reeve et al. (2014) conclude that young people increase their enjoyment/fun during sports practice and that non-conventional models need to be implemented, as they allow students to have the opportunity to make decisions within the teaching-learning process and to be able to choose the tasks which best suit their interests.

As far as we are aware, only one systematic review has been carried out (Dudley et al., 2011) related to the subject matter of this study. This research analyses the published literature on the effectiveness of physical education in promoting physical activity participation (19 studies), motor skills (four studies), as well as the enjoyment/fun of physical activity (seven studies) in children and adolescents. Of the studies analyzed in this research, six were developed in Secondary Education and one in Primary Education. Only six studies provided the prescribed plan followed by teachers: two studies developed a curriculum negotiated with study participants; two studies used one or more teaching styles of Mosston and Ashworth (1986) in the intervention, and two other studies adopted approaches based on direct or explicit teaching or a direct instruction model. Nonetheless, this study did not focus on the analysis of interventions related to the sport teaching and their effects on participants' enjoyment/fun. Furthermore, the authors did not perform a quantitative analysis of the effects of the interventions. The main finding of this study was that its authors conclude their study by highlighting the lack of quality evaluations which did not obtain accurate conclusions on the effectiveness of interventions aimed at improving students' enjoyment/fun in physical education classes using different types of pedagogical models (Dudley et al., 2011). Thus, this systematic review and meta-analysis focuses on answering research questions and analyzing in detail the available evidence (studies that have answered these research questions) (Fernandez-Chinguel et al., 2019) on the use of different sport teaching methodologies and the effects on enjoyment/fun, synthesizing the results found in these researches. In this way, it is intended to answer research questions, and serve as a basis for making decisions based on evidence. No-conventional methodologies have demonstrated their potential for achieving multiple psychological benefits, technical-tactical learning, and enjoyment of physical activity (Chu and Zhang, 2018). In addition, unconventional methodologies are associated with higher levels of confidence and fun during practice (Braithwaite et al., 2011). The study carried out can be very useful when planning physical education classes or training sessions in order to find methodologies for sports teaching that boys and girls can have fun with, and increase participation and learning in sports. Another application of the study is related to the promotion of fair play in sports practice and the use of teaching models, since high levels of enjoyment/fun are related to a high level of fair play (Cecchini et al., 2007). In addition, this research may serve to underline the importance of the methodological intervention of the teacher in creating an environment in which children can perceive the practice of sport as fun, in order to encourage them to continue their practice even outside the school setting (Coppola et al., 2020). For this reason, the questions raised in this review and meta-analysis are the following: 1. Are effective those interventions aimed at developing young people's enjoyment/fun during sport practice when using non-conventional methodologies? 2. What are the characteristics of the interventions promoting to a greater extent the development of enjoyment/fun? In this sense, the aim of this study was to analyze the effects of interventions using conventional and non-conventional sport teaching methodology on students' enjoyment/fun, through a systematic review and meta-analysis.

## Method

To perform this review and meta-analysis, we followed the Preferred Reporting Items of Systematic reviews and Meta-Analyses (PRISMA) Statement and the practical guide for systematic reviews (Urrútia and Bonfill, 2010; Moher et al., 2015; Page et al., 2021).

### Eligibility Criteria

The inclusion criteria applied in this study were as follows: (a) manuscripts had to be available in full text; (b) papers had to have been published between 2000 and 2020; (c) studies had to have an intervention programme with a control group and an experimental group; (d) interventions could be in any sport; (e) the setting of the interventions should be related to physical education classes or extra-curricular sports; (f) research had to include a pre-test and a post-test; (g) manuscripts had to be in English, Portuguese or Spanish; (h) articles could not be systematic reviews or reviews of the literature; and (i) they had to show mean and standard deviation in the results obtained in the pre-test and post-test, both in the control group and in the experimental group. The exclusion criteria were: (a) studies not related to sports education using a conventional or non-conventional methodology in an educational or educational setting; (b) research that does not compare the use of a conventional methodology with an unconventional methodology for sports education; (c) studies that do not include results related to enjoyment or fun; and (d) unpublished writings between 2000 and 2020.

Papers meeting all inclusion criteria were incorporated into the systematic review. Additionally, other manuscripts were added as they were found after analysis of the references of the papers obtained from the search. In order to reduce selection bias, each study was reviewed independently by two authors, who mutually determined whether or not the manuscripts met the inclusion criteria. In case of discrepancy, this was resolved by the third investigator.

### Search Strategy

The systematic search of the literature was conducted following the protocol suggested by the PRISMA guidelines (Page et al., 2021). The search was undertaken between 24 February and 30 May 2021 in the following databases: Web of Science; Scopus; Sportdiscus; Pubmed; Eric and Psycinfo. In the search process, four distinct blocks were defined to ensure that the studies shown were as closely related as possible to the subject matter of this systematic review. The search blocks were as follows: (a) teaching games OR teaching sports OR sport pedagogy; (b) sports training OR physical education; (c) fun OR enjoyment OR diversion OR entertainment OR satisfaction; (d) intervention OR experimental OR quasi-experimental OR randomized controlled trial. As an example, for the SportDiscus database the search phrase was: (teaching games OR teaching sports OR sport pedagogy) AND (sports training OR physical education) AND (fun OR enjoyment OR diversion OR entertainment OR satisfaction) AND (intervention OR experimental OR quasi-experimental OR randomized controlled trial). This search phrase was entered only in English. For the development of the search phrase, the thematic blocks were first established. Then, after searching the selected databases and articles related to the topic of study, the terms were added in each thematic block, as well as their synonyms and related terms.

### Study Selection and Data Collection Process

Once the search was completed, an analysis of both the title and the abstract was run in order to find those papers most directly connected to the subject matter and to eliminate or exclude those which did not meet the inclusion criteria. After the screening process, the papers were selected for subsequent data collection. The number of papers meeting all the inclusion criteria was 11. However, it is worth mentioning that the study by Cecchini et al. (2007) considered two experimental groups.

### Risk of Bias Assessment

To assess the risk of bias, the Physiotherapy Evidence Database (PEDro) scale (Maher et al., 2003) was used. Its main objective is to analyse the quality of interventions in different studies, mainly in randomized controlled trials. On the other hand, the GRADE (Grading of Recommendations Assessment, Development, and Evaluation) approach (Balshem et al., 2011) was used to assess the quality of evidence. This approach features a four-level scale (“very low,” “low,” “moderate” and “high”). The quality of evidence for a study is downgraded when there is poor applicability of the evidence, inconsistency or inaccuracy, or publication bias.

### Data Collection

Considering the PRISMA statement, information regarding participants, comparisons, intervention, study design and outcomes (PICOS, Urrútia and Bonfill, 2010) was included (Moher et al., 2009). The studies were reviewed independently by two authors of this research. In case of doubt, this was resolved by the other investigator.

### Statistical Analysis

In the meta-analysis carried out, a random-effects model was used to measure the effect of interventions on enjoyment during sport. The effect size was calculated using means and standard deviations before and after the intervention applied to participants (Morris and DeShon, 2002). For the meta-analysis, the magnitude of Cohen's d was specified as follows: (a) “large” for values above 0.8, (b) “moderate” for scores between 0.5 and 0.8 and (c) “small” for values between 0 and 0.5. Heterogeneity was assessed by calculating the following statistics: (a) Tau^2^, for the calculation of between-study variance, (b) Chi^2^ and (c) *I*^2^, which is a transformation of the H-statistic, used to determine the percentage of variation caused by heterogeneity (Higgins and Thompson, 2002; Higgins et al., 2003). The most common classification of *I*^2^ considers values above 50% as high heterogeneity, values between 25 and 50% as moderate and values below 25% as small (Higgins and Thompson, 2002). The Review Manager 5.4 tool was used to perform all analyses (Nordic Cochrane Centre the Cochrane Collaboration, 2020).

## Results

### Study Selection

**Figure 1:**
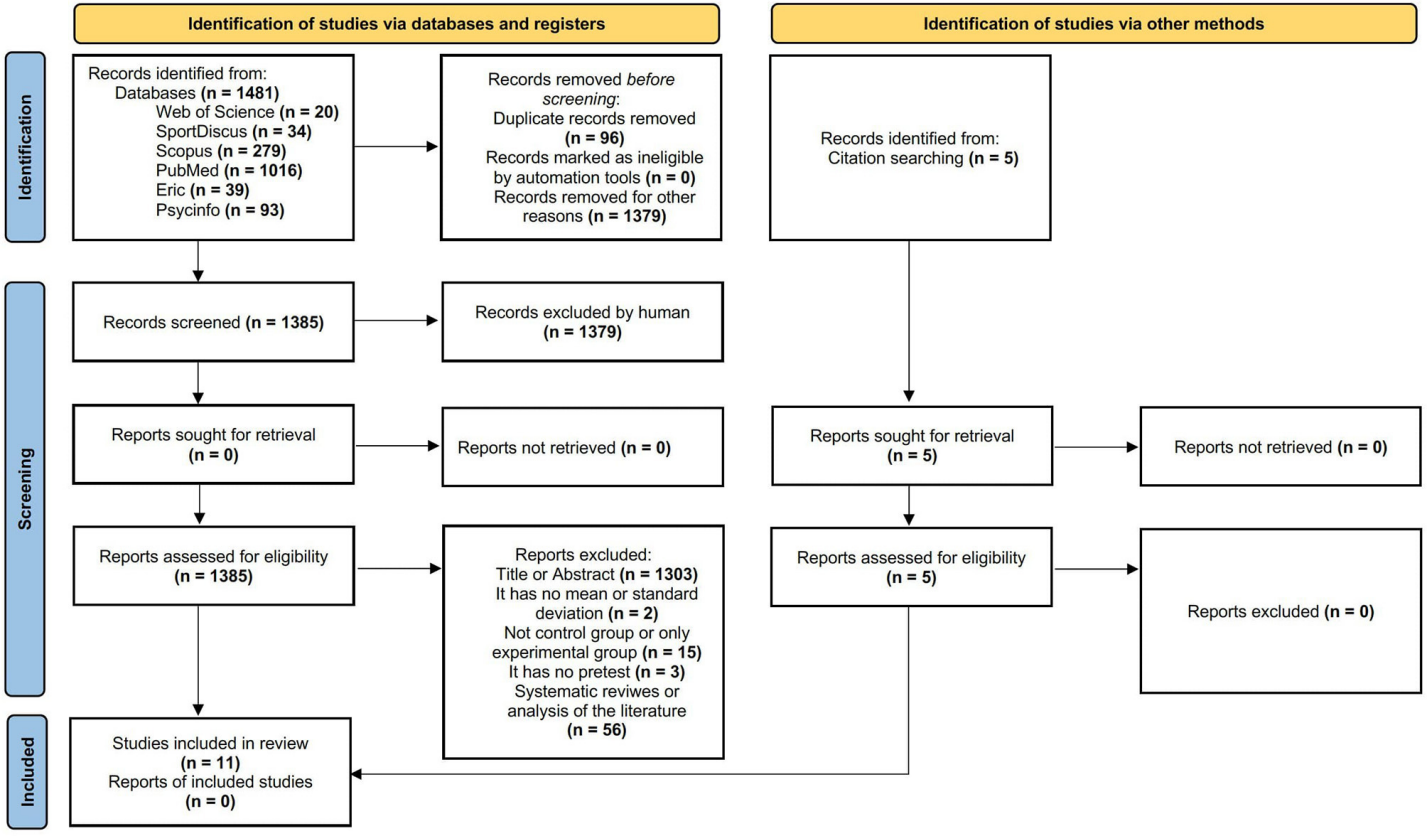
Flowchart of the systematic review process according to the PRISMA protocols declarations.

After searching the databases, a total of 1,481 documents were obtained, with the addition of five more which were identified in the reference lists of the articles found in the databases. Once the duplicates (96) were removed, a total of 1,385 articles remained, out of which 1,379 were excluded after applying the inclusion/exclusion criteria. Following this process, in the end, 11 articles were included in this systematic review (see Figure 1). However, some studies that might appear to meet the inclusion criteria were excluded. In this sense, the research of Myers et al. (2019) was not included because it did not directly relate to the subject under study, while the study by Yu et al. (2018) was discarded as a systematic review. Two reviewers independently analyzed the studies in the selection process, and inter-observer agreement was calculated, yielding a reliability of 97.9% (Thomas and Nelson, 2007).

### Risk of Bias

Table 1 shows the results of the risk of bias of those studies which have been included in this study. Moreover, it should be noted that, for the risk of bias assessment, and for the quality of evidence assessment, the studies were reviewed independently by two authors of this research. In case of doubt, this was resolved by the other investigator.

**Table 1:** Risk of bias according to the PEDro Scale.

**Study**	**1**	**2**	**3**	**4**	**5**	**6**	**7**	**8**	**9**	**10**	**11**	**Total**
**Response to each item level of evidence**												
Casado-Robles et al. (2020)	Y	N	Y	Y	Y	N	N	Y	Y	Y	Y	7
Cecchini et al. (2003)	Y	N	Y	Y	Y	N	N	Y	Y	Y	Y	7
Cecchini et al. (2007)	Y	N	Y	Y	Y	N	N	Y	Y	Y	Y	7
Coppola et al. (2020)	Y	N	Y	Y	Y	N	N	Y	Y	Y	Y	7
Cuevas et al. (2016)	Y	N	Y	Y	Y	N	N	Y	Y	Y	Y	7
Gil-Arias et al. (2020)	Y	N	Y	Y	Y	N	N	Y	Y	Y	Y	7
Lis-Velado and Carriedo (2019)	Y	N	Y	Y	Y	N	N	Y	Y	Y	Y	7
Spittle and Byrne (2009)	Y	N	Y	Y	Y	N	N	Y	Y	Y	Y	7
Viciana et al. (2020)	Y	N	Y	Y	Y	N	N	Y	Y	Y	Y	7
Wallhead and Ntoumanis (2004)	Y	N	Y	Y	Y	N	N	Y	Y	Y	Y	7
Wallhead et al. (2014)	Y	N	Y	Y	Y	N	N	Y	Y	Y	Y	7

*Y, criterion fulfilled; N, criterion not fulfilled; 1, eligibility criteria were defined; 2, participants were randomly distributed into groups; 3, the assigned was concealed; 4, the groups were similar before of the intervention (at baseline); 5, all participants were blinded; 6, therapists (teachers) who conducted the intervention were blinded; 7, there was blinding of all evaluators; 8, the measures of at least one of the fundamental outcomes were attained from more than 85% of the participants initially; 9, “intention to treat” analysis was conducted on all participants who received the control condition or treatment as assigned; 10, the findings of statistical comparisons between groups were reported for at least one fundamental outcome; 11, the study gives variability and punctual measures for at least one fundamental outcome; total score: each satisfied item (except the first) adds 1 point to the total score*.

Table 1 shows the risk of bias of the 11 articles included in the meta-analysis according to the PEDro Scale. The total study scores were equal across the eight manuscripts (seven points). In terms of quality of evidence, the GRADE guidelines were followed. The studies were not randomized clinical trials, so the rating started in low quality. Regarding the risk of bias of the results, it was not considered to lower the grade because much of the information came from studies with low risk of bias. However, the quality of evidence was downgraded on one occasion due to a high degree of heterogeneity (Tau^2^ = 0.13; Chi^2^ = 76.27, df = 11; *p* < 0.00001; *I*^2^ = 86%). Regarding indirectness, the rating was not lowered, since the evidence of the meta-analysis performed answered the questions of the review. As for the imprecision, since a sufficient number of people were included (*n* = 938), and since the confidence interval was not large (95% CI from 0.48 to 0.96), it was decided not to lower the score. Finally, no publication bias was detected, so the rating was not lowered for this reason. In addition, although the effect size was moderate (0.72), the rating was not upgraded. For this reason, the quality of evidence, according to the GRADE guidelines, was “very low,” which was defined as “we have very low confidence in the estimate of the effect: the true effect is likely to be substantially different from the estimate of the effect” (Balshem et al., 2011, p. 404).

### Characteristics of Studies

Table 2 outlines the characteristics of participants, duration, instrument, and protocol of the interventions, including the sports played. In addition, Table 3 details the objectives, the intervention programmes of each study, as well as the main findings of the research. Table 2 describes the characteristics of the studies included in this systematic review. It should be pointed out, on the one hand, that there was a total of 1,806 participants, which were distributed in such a way that 868 subjects formed the control groups and 938 the experimental groups.

**Table 2:** Participants' characteristics, duration, data collection and sample selection, other variables measured, and instrument included in the systematic review and meta-analysis.

**Study and Country**	**Study design**	***N* (gender)** ***N* (control group and experimental group)**	**Age and educational level/context**	**Duration of the study**	**Data collection and sample selection**	**Instruments to measure enjoyment/fun**	**Other variables and measure instruments**
Casado-Robles et al. (2020) and Granada (Spain)	Quasi-experimental	114 NR^*^ NR^*^. Only 99 participated Control G. (44) Experimental G. (55)	M = 14.0 SD = 1.1 2nd and 4th year of Secondary Education^1^	6 weeks 12 sessions 2 sessions/week 1 h/session	Participants completed pre-test measures and then participated in their classes (SE and traditional teaching). Post-test measures were completed in the last class of each methodology. Cluster Randomized	Multidimensional Sportspersonship Questionnaire (CMD) by Iturbide-Luquin and Elosua-Oliden (2017) This questionnaire consists of 21 items divided into five dimensions: (a) Enjoyment; (b) Respect; (c) Commitment; (d); (e) Participation. All of them are evaluated with a 5-point Likert scale ranging from 1 “Strongly disagree” to 10 “Strongly agree”	Anthropometry. They were recorded following the International Standards for Anthropometric Assessment (Stewart et al., 2011) Perceived habitual physical activity. The perceived habitual physical activity of the participants was assessed using the PACE (Physician-based Assessment and Counseling for Exercise) questionnaire, in its version adapted and validated to the Spanish context for adolescents by Martínez-Gómez et al. (2009) Intention to be physically active. Participants' intention to be physically active was assessed using the Measure of Intention to be Physically Active (MIFA) questionnaire, in its version adapted and validated to the Spanish context by Moreno et al. (2007) Objective levels of physical activity and sedentary behavior. Levels of habitual physical activity and sedentary behavior were assessed objectively using a GT3X accelerometer (ActiGraph, LLC, Pensacola, FL, USA)
Cecchini et al. (2003) and North of Spain	Quasi-experimental	142 (73 women) (69 men) Control G. (70) Experimental G. (72)	M = 12.5 NR^*^ 1st year of Secondary Education	10 sessions 1 h/session	Participants completed the pre-test measures and then participated in their classes (SE and traditional teaching). Post-test measures were completed in the last class of each methodology. Cluster Randomized	Fair Play Attitudes Scale (Cruz et al., 1996) This questionnaire consists of 23 items divided into three dimensions: (a) Hard play; (b) Victory and (c) Fun. All of them are evaluated with a 5-point Likert scale ranging from 1 “Strongly disagree” to 5 “Strongly agree”	Self-control. It was measured through the Child and Adolescent Self-Control Questionnaire (CACIA) (Capafons and Silva, 1998).
Cecchini et al. (2007) and Asturias (Spain)	Quasi-experimental	186 (94 women) (92 men) Control G. (61) Experimental G.A (63) Experimental G.B (62)	M = 13.6 SD = 0.30 2nd year of Secondary Education	2 months	Participants completed the pre-test measures and then participated in their classes (Hellison Programme and traditional teaching). Post-test measures were completed in the last class of each methodology. Cluster Randomized	Fair Play Attitudes Scale (Cruz et al., 1996) This questionnaire consists of 23 items divided into three dimensions: (a) Hard play; (b) Victory and (c) Fun. All of them are evaluated with a 5-point Likert scale ranging from 1 “Strongly disagree” to 5 “Strongly agree”	Self-control. It was measured through the Child and Adolescent Self-Control Questionnaire (CACIA) (Capafons and Silva, 1998).
Coppola et al. (2020) and Italy	Quasi-experimental	66 (35 women)(31 men) Control G. (32) Experimental G. (34)	M = 8.6 SD = 0.4 4th year of Primary Education	22 weeks 1 session/week 1 h/session	Participants completed pre-test measures and then participated in their classes (Tactical Model and traditional teaching). Post-test measures were completed in the last class of each methodology. Cluster Randomized	Physical Activity Enjoyment Scale (PACES) questionnaire (Carraro et al., 2008) This questionnaire consists of 16 items. All of them are evaluated with a 5-point Likert scale ranging from 1 “Strongly Disagree” to 5 “Strongly Agree”	This study only measured enjoyment
Cuevas et al. (2016) and Spain	Quasi-experimental	86 (49 women) (37 men) Control G. (43) Experimental G. (43)	M = 15.65 SD = 0.78 4th year of Secondary Education	19 sessions 55 min/twice a week	Participants completed the pre-test measures and then participated in their classes (DE and traditional teaching). Post-test measures were completed in the last class of each methodology. Cluster Randomized	Spanish version (Balaguer et al., 1997) of the original Intrinsic Satisfaction Classroom Scale (ISC) (Duda and Nicholls, 1992) This questionnaire consists of 16 items divided into two dimensions: (a) Satisfaction/Enjoyment; (b) Boredom. All of them are evaluated with a 5-point Likert scale ranging from 1 “Strongly disagree” to 5 “Strongly agree”	Motivational regulation. It was measured through the Questionnaire for the Evaluation of Motivation in Physical Education (CMEF) (Sánchez-Oliva et al., 2012) Intention to be physically active. It was measured through the Intention to Be Physically Active Scale (IPAS) (Hein et al., 2004), but the Spanish version of the Spanish version was used (Moreno et al., 2007)
Gil-Arias et al. (2020) and Southeast Spain	Quasi-experimental	292 (140 women)(152 men) Control G. (144) Experimental G. (148)	M = 10.41 SD = 0.49 5th year of Primary Education	8 weeks 16 sessions 50 min/twice a week	Participants completed pre-test measures and then participated in their classes (Hybrid TGfU/ED and direct instruction). Post-test measures were completed in the last class of each methodology. Cluster Randomized	Spanish version (Sicilia et al., 2014) of Physical Activity Class Satisfaction Questionnaire (Cunningham, 2007) This questionnaire consists of 45 items divided into nine dimensions, although in this study only (a) Fun/Enjoyment; (b) Interaction with others; (c) Experiences of mastery; (d) Experience of fun were evaluated. All of them are evaluated with an 8-point Likert scale ranging from 1 “Strongly Disagree” to 8 “Strongly Agree”	Autonomy support. The Spanish version (Conde et al., 2010) of the Autonomy Support Strategies Questionnaire of coaching strategies (Conroy and Coatsworth, 2007) Satisfaction of Basic Psychological Needs (BPN) To assess satisfaction with BPN, the Spanish adaptation of the BPN in exercise scale (Vlachopoulos and Michailidou, 2006), specific to the context of physical education, was used (Moreno-Murcia et al., 2008, 2009) Autonomous motivation. The Spanish version (Ferriz et al., 2015) of the Perceived Locus of Causality Questionnaire was used to provide composite scores for autonomous motivation (Goudas et al., 1994) Friendship goals. The Spanish adaptation (Méndez-Giménez et al., 2014) of the friendship relationship goals questionnaire (Elliot et al., 2006) was used
Lis-Velado and Carriedo (2019) and North of Spain	Quasi-experimental	92 (42 women)(50 men) Control G. (45) Experimental G. (47)	M = 10.38 SD = 0.55 5th year of Primary Education	3 sessions 60 min/session	Participants completed the pre-test measures and then participated in their classes (Brave League and traditional competitive system). Post-test measures were completed in the last class of each methodology. Cluster Randomized	Fair Play Attitudes Scale (Cruz et al., 1996) This questionnaire consists of 23 items divided into three dimensions: (a) Hard play; (b) Victory and (c) Fun. All of them are evaluated with a 5-point Likert scale ranging from 1 “Strongly disagree” to 5 “Strongly agree”	Goal orientations. The Spanish version (Cervelló et al., 1999) of the Perception of Success Questionnaire (POSQ) (Roberts and Balagué, 1991; Roberts et al., 1998) was used Pressure-strain and effort. Two of the four subscales of the Intrinsic Motivation Questionnaire (IMI) by McAuley et al. (1989), which was translated and validated in Spanish by Escartí and Gutiérrez (2001), were used to measure the pressure-strain and effort perceived by the students
Spittle and Byrne (2009) NR^*^	Quasi-experimental	115 (18 women) (97 men) Control G. (74) Experimental G. (41)	NR^*^ NR^*^ 2nd year of Secondary Education	10 weeks 100 min/weeks	Participants completed the pre-test measures and then participated in their classes (SE and traditional teaching). Post-test measures were completed in the last class of each methodology. Cluster Randomized	Intrinsic Motivation Inventory (IMI) (Ryan, 1982), reformulated for use in sport settings by McAuley et al. (1989) This questionnaire consists of 18 items divided into four dimensions: (a) Enjoyment/Interest; (b); Effort/Importance; (c) Perceived Competence; Pressure/Strain. All of them are evaluated with a 5-point Likert scale ranging from 1 “Strongly disagree” to 5 “Strongly agree”	Goal orientation. Goal orientations were assessed using the Task and Ego Orientation in Sport Questionnaire (TEOSQ). (Duda and Nicholls, 1992) Motivational climate. Perceived motivational climate was measured with the Perceived Motivational Climate in Sport Questionnaire (PMCSQ). (Walling et al., 1993)
Viciana et al. (2020) and Ceuta (Spain)	Quasi-experimental	123 (60 women) (63 men) Only 109 participated Control G. (42) Experimental G. (67)	NR^*^ NR^*^ 3rd year of Secondary Education	12 sessions	Participants completed the pre-test measures and then participated in their classes (SE and traditional teaching). Post-test measures were completed in the last class of each methodology. Cluster Randomized	Spanish version adapted to physical education (Baena-Extremera et al., 2012a) of the Sport Satisfaction Instrument This questionnaire consists of 8 items divided into two dimensions: (a) Satisfaction/Fun; (b) Boredom. All of them are evaluated with a 5-point Likert scale ranging from 1 “Strongly disagree” to 5 “Strongly agree”	Motivation toward Physical Education. The Spanish version of the Perceived Locus of Causality (Moreno et al., 2009) was used. Perceived physical fitness, sport competence and coordination. For these physical self-concept subscales, two questionnaires were used (a) the Spanish version of the Physical Self-perception (Moreno and Cervelló, 2005); (b) the Spanish version of the Physical Self-description (Tomás, 1998) Effort and self-improvement. The effort and improvement subscale belonging to the Spanish version of the Perceived Motivational Climate in Sport Questionnaire (PMCSQ-2). (González-Cutre et al., 2008) Relationship. The Spanish version of the Cuestionario Basic Psychological Needs in Exercise (BPNES) (Sánchez and Núñez, 2007)
							Cooperative learning and important role. The cooperative learning and important role subscales belonging to the Spanish version of the PMCSQ-2 (González-Cutre et al., 2008) Classroom climate. Classroom climate was measured with the Spanish version of the Classroom Environment Inventory (Marcelo, 1992) Sportsmanship. The Spanish version of the Multidimensional Scale of Sport Orientations (MSOS) by Martín-Albo et al. (2006) Intention to be physically active questionnaire. The Spanish version of the intention to be physically active (Moreno et al., 2007) Autonomy support. Spanish version of the BPN (Sánchez and Núñez, 2007)
Wallhead and Ntoumanis (2004) and North of England	Quasi-experimental	51 NR^*^ NR^*^ Control G. (26) Experimental G. (25)	M = 14.3 SD = 0.48 2nd year of Secondary Education	8 sessions 1 h/sesssion	Participants completed pre-test measures and then participated in their classes (SE and traditional teaching). Post-test measures were completed in the last class of each methodology. Cluster Randomized	Intrinsic Motivation Inventory (IMI) (Ryan, 1982), reformulated for use in sport settings by McAuley et al. (1989) This questionnaire consists of 18 items divided into four dimensions: (a) Enjoyment/Interest; (b); Effort/Importance; (c) Perceived Competence and (d) Pressure/Strain. All of them are evaluated with a 5-point Likert scale ranging from 1 “Strongly Disagree” to 5 “Strongly Agree”	Goal orientation. Goal orientations were assessed using the Task and Ego Orientation in Sport Questionnaire (TEOSQ). (Duda and Nicholls 1992) Perceived autonomy. Students' perceived autonomy was assessed using a 20-item questionnaire adapted to physical education by Goudas et al. (1994). The items were taken from the Academic Self-Regulation Questionnaire (ASRQ) (Ryan and Connell, 1989) and the Academic Motivation Scale (Vallerand et al., 1992) Perceptions of motivational climate. These were assessed with the Learning and Performance Orientation in Physical Education Classes Questionnaire (LAPOPECQ). (Papaioannou, 1995)
Wallhead et al. (2014) and Midwestern (United States)	Quasi-experimental	568 (310 women) (258 men) Control G. (287) Experimental G. (281)	M = 14.75 SD = 0.48 3rd year of Secondary Education	9 months 2 session/week 45 min/sesssion	It is possible that different types of activities (e.g., cooperative games vs. team sports vs. individual sports) may have caused temporary fluctuations in student motivation. Cluster Randomized	Intrinsic Motivation Inventory (IMI) (Ryan, 1982), reformulated for use in sport settings by McAuley et al. (1989) This questionnaire consists of 18 items divided into four dimensions: (a) Enjoyment/Interest; (b); Effort/Importance; (c) Perceived Competence and (d) Pressure/Strain. All of them are evaluated with a 5-point Likert scale ranging from 1 “Strongly disagree” to 5 “Strongly agree”	Type of autonomous motivation. Autonomous motivation type was assessed using the Perceived Locus of Causality Questionnaire presented by Goudas et al. (1994) NPB. The NPB Scale adapted to physical education (Ntoumanis, 2005) Intention to be physically active. The Physical Activity Intention Scale followed the model of (Fishbein and Ajzen, 2010).

NR* *, not reported*.

1 *According to the Spanish educational system*.

**Table 3:** Objectives, intervention programme, main results, conclusions, and limitations of the research included in the systematic review and meta-analysis.

**References**	**Objectives and hypotheses**	**Intervention programme**		**Main Results**	**Conclusions**	**Limitations**
		**Control Group**	**Experimental Group**			
Casado-Robles et al. (2020)	To measure the effect of a teaching unit of the Sport Education Model in physical education on sportspersonship and on the levels of regular physical activity Hypothesis: NR^*^	The students belonging to the control group played basketball during Physical Education classes following a traditional teaching method.	The students in the experimental group played basketball during Physical Education classes following the SE.	The Sport Education Model programme improved the students' sportspersonship dimension. However, there were no significant differences on the other dimensions of sportspersonship or on the students' regular physical activity levels.	A 12-session SE didactic unit increased students' desire and willingness to participate in sport competitions by applying maximum effort. However, the programme does not have a greater effect compared to the traditional model on the other dimensions of sportsmanship, nor on levels of habitual physical activity and intention to be physically active. For this reason, the effect of the programme should be tested in other contexts (e.g., with longer programme duration and/or different content)	Duration of programme (12 sessions) High number of excluded participants and, consequently, low final sample size (45 participants) Sample from a single school
Cecchini et al. (2003)	To assess the impact of Hellison's (1995) Intervention Programme for Developing Personal and Social Responsibility on fair play behaviors and on self-control Hypothesis: In a relatively short time, the implementation of this programme would generate positive changes in the opinions and behaviors related to fair-play in sport, as well as in personal self-control in other domains outside sport	The boys and girls in the control group played indoor football during physical education classes following a traditional teaching method.	The boys and girls in the experimental group practiced indoor football during Physical Education classes following the Hellison intervention programme, which is structured in five levels of responsibility. (a) respecting the rights and feelings of others; (b) being motivated; (c) autonomy; (d) helping others; (e) applying these responsibilities to other domains outside sport.	Significant improvements were observed in personal feedback, reward delay, criterial self-control, process self-control, fun-related opinions, and sporting behaviors. Decreases were observed in variables related to rough play, contact fouls and unsporting behaviors. Finally, no significant changes were found in the control group.	Physical activities can be, when properly addressed, a vehicle for effecting changes in social and moral development in children and adolescents. In addition, it is possible to successfully implement a programme of 'personal responsibility and social responsibility' in physical education classes, providing a useful tool for moral development and moral development through sport and physical activity	NR^*^
Cecchini et al. (2007)	To assess the impact of Hellison's (1995) Intervention Programme for Developing Personal and Social Responsibility on fair play behaviors and on self-control Hypothesis: Level 5 of Hellison's (1995) programme is essential for the transfer to take place	The control group students played five-a-side football during Physical Education following a Traditional teaching method	The students of the experimental groups A and B practiced indoor football following the Hellison intervention programme, which is structured in five levels of responsibility, which were worked on by group A, while group B only worked on the first four. (a) respecting the rights and feelings of others; (b) being motivated; (c) autonomy; (d) helping others; (e) applying these responsibilities to other domains.	In the experimental groups A and B, significant improvements were observed in personal feedback, reward delay, criterial self-control, process self-control, fun-related opinions, and sporting behaviors. Decreases were observed in variables related to rough play, contact fouls and unsporting behaviors. Finally, no significant changes were found in the control group.	It is possible to successfully implement a programme of “personal responsibility and social responsibility” in physical education classes, providing a useful tool for moral development through sport and physical activity	Sample. Given that there are many factors that influence transfer, such as age, mental ability personality and motivation
Coppola et al. (2020)	To analyse the effects of two different teaching approaches on students' levels of enjoyment Hypothesis: NR^*^	The boys and girls in the control group played different sports (basketball, handball, football, and volleyball) in Physical Education classes following a Traditional teaching method.	The boys and girls in the experimental group played basketball, handball, football and volleyball during physical education classes following this approach: a tactical problem, a lesson approach and an objective.	The results obtained revealed that male participants in the experimental group did not show variations in the levels of the enjoyment scale, while female participants, although starting from a lower level, had increases in the scale scores in the post-intervention, although not statistically significant.	Recent work has shown how the TGM approach is functional both for the personal development of students and for the learning of skills that can go beyond motor-sport practice (curricular and extracurricular), also affecting enjoyment	The duration and frequency of the interventions, as it was only 1 h per week
Cuevas et al. (2016)	To analyse the impact of the Sport Education Model on self-determination, motivation, frustration of basic psychological needs, enjoyment-satisfaction, boredom in Physical Education of secondary school students Hypothesis: NR^*^	The students in the experimental group, during the Physical Education sessions, played volleyball following a Traditional teaching method.	Students in the experimental group played volleyball in physical education classes, following the SE.	The results showed significant improvements in different items such as enjoyment and intrinsic motivation in favor of the Experimental Group.	Through the implementation of group work, opportunities for social interaction between team members are provided; however, these opportunities may be significantly reduced by other classmates who are members of opposing teams	Sample size, sample selection method and duration of the intervention
Gil-Arias et al. (2020)	To investigate the motivational outcomes of primary school children participating in an invasion game unit through two pedagogical models: a hybrid TGfU/Sport Education unit or a Direct instructions unit Hypothesis: Boys and girls participating in the hybrid TGfU/SE unit will report higher levels of autonomy support compared to boys and girls participating in the direct instruction unit Children taught through the hybrid TGfU/SE model will report higher scores on all post-intervention variables compared to pre-intervention children participating in the direct instruction model	The boys and girls belonging to the control group practiced different invasion sports in Physical Education following a traditional teaching method.	The boys and girls in the experimental group played different invasion sports through hybrid TGfU/ SE sessions during Physical Education lessons.	Significant differences in student motivation were observed for both boys and girls who participated in the TGfU/SE hybrid unit.	A hybrid TGfU/SE unit can be implemented in a physical education context as it can lead to significant improvements in students' self-determined motivation when compared to a direct instruction unit	Only the effects of a hybrid TGfU/SE season were examined
Lis-Velado and Carriedo (2019)	To analyse the impact of the innovative Brave League on goal orientations, fair play, effort, and pressure-stress. Hypothesis: NR^*^	The students belonging to the control group played indoor football during the physical education sessions following a traditional competitive system (points, ranking, etc.).	The students in the experimental group practiced indoor football during Physical Education following a competitive system based on the innovative format of the Brave League, where fair play behaviors determine the order in the ranking.	The results attained suggest that competitive formats could have a positive impact on task orientation, enjoyment, and effort during sport competitions.	The proximity in time between the pre and post measurement The Brave League format can produce changes in a relatively short period of time in the task orientation and effort of the participants without altering the perceived enjoyment during the competition	The number of participants The short time spent on the intervention in each of the groups
Spittle and Byrne (2009)	To investigate the influence of the SE on student motivation (intrinsic/extrinsic motivation, goal orientations and perceived motivational climate) in secondary school Physical Education Hypothesis: Enjoyment, perceived competence, effort, task orientation and mastery-oriented motivational climate would increase significantly. In addition, pressure/strain, ego-orientation, and performance-oriented climate would decrease due to SE. A task climate would be related to increased intrinsic motivation, and a performance climate would be related to ego-goal orientation and decreased intrinsic motivation	The students in the control group played soccer and field hockey during the physical education sessions, following a traditional teaching method	Students in the experimental group, in physical education classes, played soccer, hockey and “code football” a combination of Australian Rules Football, Gaelic football and played soccer, field hockey and “code soccer” (combination of Australian Rules Football, Gaelic soccer and touch soccer) following the Sports Education model	There was a significant difference between conditions on changes in perceived competence, task orientation and mastery climate. There were no significant differences in enjoyment/fun, effort, and pressure.	SE was more successful in maintaining high levels of intrinsic motivation, task orientation and climate of mastery than the traditional condition	Existence of several confounding variables that were not controlled for or measured; consequently, it is difficult to conclude a causal relationship between the independent variable and the dependent variables
Viciana et al. (2020)	To analyse the effect of a physical education-based and Sport education programme on personal and interpersonal variables, on the social environment and on the predisposition to acquire positive habits and autonomy Hypothesis: NR^*^	The boys and girls belonging to the control group practiced volleyball following a Traditional teaching method during Physical Education classes, combining tasks with reduced game situations and Direct instructions methodologies.	The boys and girls in the experimental group played volleyball during the P.E. sessions following the SE based on games and competitions of volleyball game situations.	Participants in the Experimental Group had a statistically significant increase in scores on the following dimensions: personal (self-determined motivation toward physical education, satisfaction/enjoyment toward sport and physical self-concept); interpersonal (relationship with others, cooperative learning, and important role within the group); social (Physical Education classroom environment and sportspersonship); and autonomy and habit acquisition (support for autonomy and intention to be physically active) when compared to the Control Group.	It is very useful to design effective programmes that allow for positive results in terms of interpersonal, social and habit acquisition, fun, motivation, and autonomy to improve pupils' citizenship education	Issues beyond the control of the researchers Lack of personal resources
Wallhead and Ntoumanis (2004)	To analyze the influence of a SE intervention program on students' motivational responses in a high school physical education setting Hypothesis: Students in the SE group would report a greater increase in enjoyment, perceived effort, and perceived competence than those in the traditional curriculum group. Changes in SE students' perceptions would significantly predict the intervention dependent variables of students' enjoyment, effort and competence	The boys and girls in the control group, during the Physical Education sessions, played basketball following traditional teaching	The boys and girls in the experimental group, in physical education classes, played basketball following the SE	The SE increases the perception of a climate of task involvement and perceived autonomy and in doing so, increase motivation and enjoyment of high school students toward physical education	The SE has many structural features that have the potential to foster more adaptive motivational responses from students by creating an environment that allows for self-improvement, choice, and equity for students	One was the size and composition of the intervention sample. With only two groups of boys in its design, this study cannot be easily generalized to girls participating in the SE There is also the possibility of bias, as the researcher acted as a teacher and was aware of the aims of the study
Wallhead et al. (2014)	To examine the effect of the SE in high school on student motivation in Physical Education Hypothesis: Participants completed pre-test measures and then participated in their classes (SE and multi-activity programme). Post-test measures were completed in the last class of each methodology	The students in the control group played, during physical education sessions, volleyball, badminton, soccer, ultimate, cooperative games, handball, and basketball following a multi-activity model	Students in the experimental group, field hockey, volleyball, handball, basketball, during the physical education sessions, following SE	The results showed that the SE generates a greater enjoyment of the program in students compared to the students of the multi-activity model	SE facilitates more internalized forms of student motivation, but without the provision of an appropriately designed extra-curricular outlet, the potential may drop considerably	SE may not have used pedagogies that maximize support for full autonomy

NR* *, not reported*.

### Interventions

Table 2 shows the protocol used in all studies, both in experimental and control groups. As for the control groups, most of the studies involved traditional teaching (Cecchini et al., 2003, 2007; Wallhead and Ntoumanis, 2004; Spittle and Byrne, 2009; Cuevas et al., 2016: Coppola et al., 2020; Viciana et al., 2020). The control groups of Casado-Robles et al. (2020) and Gil-Arias et al. (2020), received the lessons through direct instruction, while the control group of Lis-Velado and Carriedo's (2019) study conducted a traditional competitive system. Finally, the study by Wallhead et al. (2014) received the lessons through multiactivity program.

Concerning the experimental groups, both in the study by Cuevas et al. (2016), and in those by Wallhead and Ntoumanis (2004), Spittle and Byrne (2009), Wallhead et al. (2014), Casado-Robles et al. (2020), and Viciana et al. (2020), the classes were taught using the SE. On the contrary, the studies by Cecchini et al. (2003) and Cecchini et al. (2007) worked on the basis of the Hellison Programme 1995. The experimental groups of the Lis-Velado and Carriedo (2019) and Coppola et al. (2020) studies were taught through a tactical game model and Brave League, respectively. Finally, the experimental group in the Gil-Arias et al. (2020) research was taught using a hybrid model between the TGfU and the SE. Table 3 shows the objectives, intervention programmes and main results of the articles included in this systematic review.

### Outcomes Measure

**Figure 2:**
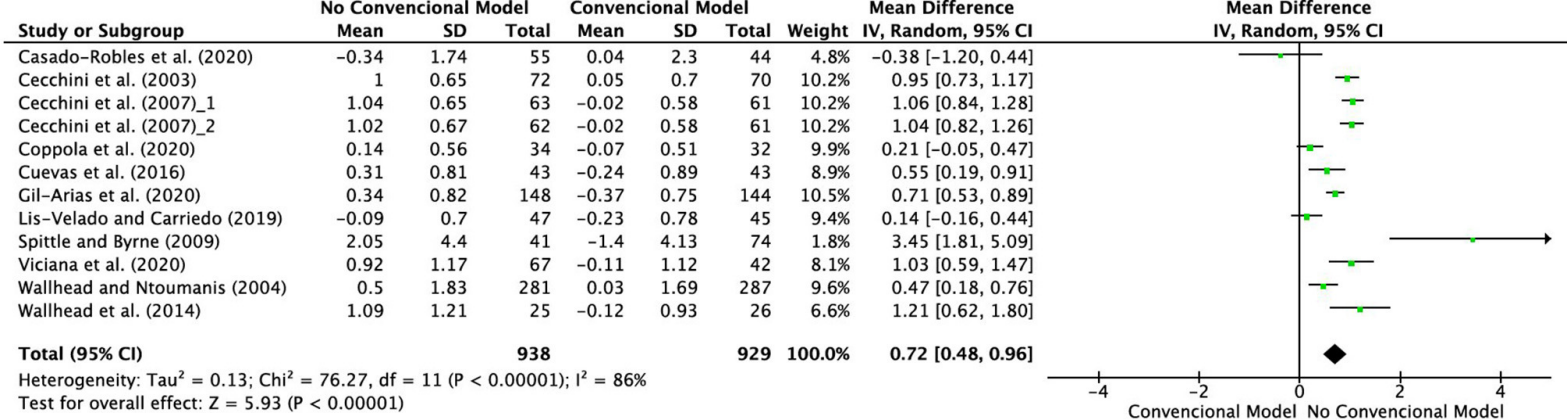
Results of the meta-analysis of the effects of interventions on fun in sport practice.

Figure 2 depicts that 11 studies obtained improvements in enjoyment during the development of sport practice through non-conventional methods. The differences were significant in five studies (Cecchini et al., 2003, 2007; Wallhead and Ntoumanis, 2004; Spittle and Byrne, 2009; Wallhead et al., 2014; Cuevas et al., 2016; Gil-Arias et al., 2020; Viciana et al., 2020). In additionno significant differences were observed in the studies conducted by Lis-Velado and Carriedo (2019) and Coppola et al. (2020). Finally, no improvements in favor of the experimental group, i.e., the non-conventional model, were observed in the study performed by Casado-Robles et al. (2020). The total effect size was 0.72, with a 95% CI from 0.48 to 0.96, which, following the proposed classification, was a moderate effect size. However, the level of heterogeneity was large: Tau^2^ = 0.13; Chi^2^ = 76.27, df = 11 (*p* < 0.00001); *I*^2^ = 86%; test for total effect: Z = 5.93 (*p* < 0.00001).

## Discussion

The aim of this study was to analyze the effects of interventions using conventional and non-conventional sport teaching methodology on students' enjoyment/fun, through a systematic review and meta-analysis. The main result was that, in general, the interventions implemented in the studies analyzed showed significant improvements. Thus, this significant increase can be considered moderate, according to the overall effect size (0.72, with a 95% CI from 0.48 to 0.96 with *p*-value < 0.00001). This is consistent with previous studies that reported that the use of no-conventional methodologies promoted enjoyment/ fun during sports practice in boys and girls (López and Castejón, 2005; MacPhail et al., 2008; Perlman, 2010). However, due to the high heterogeneity (*I*^2^ = 86%) and the low quality of the evidence, the interpretation of the results obtained in the meta-analysis should be considered cautiously (Balshem et al., 2011), and, for that reason, any estimate of effect is very uncertain (Guyatt et al., 2008). In addition, since the interventions were administered to whole classes and not to individual students, the unit of statistical analysis should have been the class, so, for that reason, the results might be biased (Li et al., 2017). Therefore, more studies are needed on the effects of sport education on the enjoyment/fun of students.

The study by Cecchini et al. (2003) obtained a large effect size (0.95, with 95% CI from 0.73 to 1.17) in favor of the experimental group, as did the studies by Cecchini et al. (2007), which also reached a large effect size (1.06, with 95% CI from 0.84 to 1.28 and 1.04, with 95% CI from 0.82 to 1.26, respectively) (see Figure 2). It is worth underlining that, in these investigations, the experimental groups were taught with the Hellison Programme 1995, which emphasizes individual decision-making, group meetings and reflection, focusing the responsibility on the students, who are gradually achieving a greater degree of autonomy (Cecchini et al., 2003). This model is structured in five levels of responsibility: (a) respecting the rights and feelings of others; (b) being motivated; (c) autonomy; (d) helping others; (e) applying these responsibilities to other domains outside of sport. Similarly, both the studies by Spittle and Byrne (2009) and Wallhead et al. (2014), obtained a large effect size (3.45 with a 95% CI of 1.81–5.09 and, 1.21 with a 95% CI of 0.62–1.80, respectively). In that case, the experimental groups received their physical education classes through the SE. On the other hand, with an effect size of 1.03 and with a 95% CI from 0.59 to 1.47, in the study carried out by Viciana et al. (2020) the boys and girls in the experimental group were taught following the Game-based SE and volleyball game situations competitions, while those in the control group were taught following traditional volleyball teaching. Meanwhile, both the studies by Cuevas et al. (2016) and Gil-Arias et al. (2020), obtained a moderate effect size, where the experimental groups were delivered their physical education classes through the SE and a hybrid between TGfU and SE, respectively. Also, the study conducted by Wallhead and Ntoumanis (2004) obtained a small effect size in favor of the experimental group, who developed their physical education classes through the SE. This data is consistent with the fact that many physical education interventions using the SE (Alexander et al., 1993; Perlman, 2010). So, this model has demonstrated its potential for achieving multiple benefits, including increased enjoyment of physical activity and sport (Chu and Zhang, 2018). It seems that this increased enjoyment/fun is related to students having fun with this model because they learn sports skills as well as personal and social skills (Alexander and Luckman, 2001) and because boys and girls work in cooperative groups (Hastie, 1996). Furthermore, the study by Coppola et al. (2020) showed a small effect size in favor of the experimental group using a tactical game model, which is consistent with those obtained by Schembri et al. (2021) Similarly, the research by Lis-Velado and Carriedo (2019) showed a small effect size in favor of the experimental group, where the experimental group implemented the physical education classes through the Brave League. Finally, out of all the studies included in the systematic review and meta-analysis, only the study by Casado-Robles et al. (2020) presented results in favor of the control group.

The duration of the studies ranged from ~3 to 22 h. These differences in the duration of the interventions seem to indicate that the benefits obtained by the experimental groups may not be due to the duration of the interventions. For this reason, regardless of the duration of the intervention, it seems advisable to use non-conventional models in sport teaching with the aim of increasing the enjoyment of students, which is in line with the findings of Batez et al. (2021).

As it has been proven, the experimental groups in the studies carried out by Wallhead and Ntoumanis (2004), Spittle and Byrne (2009), Wallhead et al. (2014), Cuevas et al. (2016), Gil-Arias et al. (2020), and Viciana et al. (2020), conducted their physical education classes using the SE, and an increase in enjoyment was observed in comparison with the control groups. Based on the data obtained, the implementation of the SE significantly promotes enjoyment/fun, with what seems to be a desirable model to increase enjoyment/fun of boys and girls during the practice of sports. In this sense, Carlson and Hastie (1997) consider that the SE can help teachers to improve students' motivation, as it encourages socialization, decision making and, in addition, favors enjoyment in competitive situations where effort levels are strongly valued. In this regard, Cecchini et al. (2014) deem important for the teacher to promote a fun atmosphere where the effort and personal progress of the student is valued, which will be reflected in the intention of the students to be physically active.

Sport is often perceived as a setting which encourages fun and enjoyment, yet it is increasingly common to observe that young people, along with their teachers/coaches, focus more on achieving victories than on instilling values such as fun in children, which may be primarily due to the teaching model used (Carron and Brawley, 2008). In the study performed by Lis-Velado and Carriedo (2019), it was noticed that the Brave League fostered more enjoyment than traditional competition. This study implemented a competitive system based on the innovative, where fair play behaviors determine the order in the ranking. It should be noted that the adoption of competition ranking systems is not based solely on match results, but, taking into account aspects of sporting behavior and fair play, can motivate students and increase enjoyment and fun (Ortega et al., 2014), since an excessive emphasis on the outcomes of competitive sports would be negatively related with enjoyment of the game (Cruz et al., 1996). Thus, Buelens and Poelmans (2004) suggest that enjoyment is connected to notions of participation and integration within a team or group. Furthermore, Kirk (2005) also suggests that the focus of young people's attention should tend to be on fun and enjoyment. In the study performed by Coppola et al. (2020) the practical task focused on the development of movements and skills related to the tactical problem of the lesson. At the end of the class, he dedicated a questioning time used to verify how the students focused on the tactical problem of each lesson and what strategies they proposed to solve it (Mitchell et al., 2013). In this sense, the tactical approach and the TGfU promote fun and enjoyment among boys and girls (López and Castejón, 2005) and are related to constructivist and situated learning theories (Kirk and MacPhail, 2002), where the student's knowledge construction takes place in games, solving problems, and reflection (Forrest, 2015).

It is also worth mentioning that several investigations such as those by Wang et al. (2002), Papaioannou et al. (2006), and García-Bengoechea et al. (2010) relate enjoyment and increased physical activity and participation. Furthermore, Barkoukis et al. (2010) report that a learning-oriented atmosphere has a direct relationship with enjoyment, while Garn and Cothran (2006) underline that enjoyment seems to be a key factor in physical education classes. Moreover, Miller et al. (2005) reinforce this idea when they consider that in Physical Education classes, enjoyment is an important element of motivation when it comes to students facing the sessions proposed by the teacher. This is important, because when students are intrinsically motivated, they show interest in an activity, they experience enjoyment and feelings of competence and control (Deci and Ryan, 1985). This issue has been highlighted in the results obtained in this meta-analysis, since in most of the studies it was found that in physical education sessions in which non-conventional methodologies were used, students showed a significant increase in enjoyment, and this could be due to the fact that boys and girls felt that they were the protagonists of the teaching-learning process (Cecchini et al., 2014), and to the fact develop a self-referenced competence or gain mastery of a task (Treasure and Roberts, 2001). In this sense, in the sports education model, the students gradually assume greater responsibility, while teachers relinquish traditional up-front direct teaching roles (Alexander and Luckman, 2001). The teacher, in this way, acts as a facilitator through a series of student-centered learning strategies (Wallhead and Ntoumanis, 2004). Yet, this was not the case when they were taught in a traditional, non-student-centered way. Continuing along these lines, in non-conventional methods a wide variety of modified games or reduced situations are posed, in which students have to analyse the situation and execute the actions (Serra et al., 2011), which generates both cognitive engagement and fun (Montero, 2017). Another aspect also considered in non-conventional methods, which promotes fun and enjoyment among boys and girls, is the contextualization of the games, i.e., that they are close to the sport they are playing (López and Castejón, 2005). In view of the above, and considering the results of this study, it seems advisable to use non-conventional methods for teaching and practicing sports in order to increase the enjoyment/fun of boys and girls, which may increase their participation and learning (Côté, 1999; Côté and Hay, 2002; Hassinger-Das et al., 2017).

This systematic review with meta-analysis has some limitations. One of them relates to the fact that the literature search was limited to three languages: Spanish, English, and Portuguese; and was limited only to articles available in full text Another limitation to consider is the high heterogeneity shown in the meta-analysis, which suggests that the interpretation of the results should be approached cautiously. Furthermore, future research could focus on the impact of the TGfU and of the SE on enjoyment, as there is a lot of research concerning the impact of these approaches on motivation, but little research on enjoyment. Another future investigation, due to the scarce studies found in this review, could be the analysis of the influence of the methodology used in sport teaching in other contexts, such as, for instance, in extracurricular sport in schools and sports clubs. Future studies could also focus on clarifying the concepts of enjoyment and fun and their relationship to aspects such as intrinsic motivation. In addition, future research should also focus on the development of more powerful tools for assessing the quality of studies, both quantitative and qualitative and with different methodological designs.

In order to answer the questions of the review presented at the beginning of the manuscript, it should be said that interventions based on non-conventional methodologies have been shown to be effective for the development of enjoyment/fun among young people. In addition, in terms of the characteristics of the interventions that led to an improvement in enjoyment/fun, the results showed that interventions based on model of sports education achieved a significant increase. The findings of this study could be very useful and of practical application, mainly for physical education teachers and, although the studies included in this research belong to the educational field, it could also be interesting for coaches, when planning their classes or training sessions in order to look for methods and strategies where children have fun and enjoy themselves, since, at this age, learning while enjoying sports practice should be the most important thing.

## Conclusions

Sport teaching models can have an impact on students' enjoyment/fun during physical education lessons. In this respect, the use of non-conventional models increases students' enjoyment/fun, which can lead to greater learning. Therefore, the methodology used by the teacher becomes a key and fundamental issue. It is worth remarking that the SE stands out when it comes to increasing students' enjoyment/fun. Finally, it seems that the traditional methodology does not manage to promote the enjoyment/fun of boys and girls in sports practice.

## Data Availability Statement

The original contributions presented in the study are included in the article/supplementary material, further inquiries can be directed to the corresponding author/s.

## Author Contributions

MR put forward the review and meta-analysis idea, developed the inclusion and exclusion criteria, organized the article structure, selected the databases to be searched, developed the search string, searched the relevant literature, read the literature, and identified the articles included in this review and meta-analysis, and wrote the draft manuscript. FG assisted in conceptualizing the original question, reviewed and edited the drafted manuscript and provided critical guidance for integrating the introduction, results, and discussion topics. MA, MR, and FG contributed to identification of discussion topics for improving the interpretation of the meta-analysis, reviewed, and edited the manuscript. All authors contributed to the article and approved the submitted version.

## Conflict of Interest

The authors declare that the research was conducted in the absence of any commercial or financial relationships that could be construed as a potential conflict of interest.

## Publisher's Note

All claims expressed in this article are solely those of the authors and do not necessarily represent those of their affiliated organizations, or those of the publisher, the editors and the reviewers. Any product that may be evaluated in this article, or claim that may be made by its manufacturer, is not guaranteed or endorsed by the publisher.
